# Bioreactor-Controlled Physoxia Regulates TGF-β Signaling to Alter Extracellular Matrix Synthesis by Human Chondrocytes

**DOI:** 10.3390/ijms20071715

**Published:** 2019-04-06

**Authors:** Holger Jahr, Seval Gunes, Annika-Ricarda Kuhn, Sven Nebelung, Thomas Pufe

**Affiliations:** 1 Institute of Anatomy and Cell Biology, University Hospital Aachen, 52072 Aachen, Germany; sevalguness@gmail.com (S.G.); annikakuhn.kuhn@alumni.maastrichtuniversity.nl (A.-R.K.); tpufe@ukaachen.de (T.P.); 2 Department of Orthopaedic Surgery, Maastricht University Medical Centre+, 6229 HXMaastricht, The Netherlands; 3 Department of Diagnostic and Interventional Radiology, Aachen University Hospital, 52072 Aachen, Germany; snebelung@ukaachen.de

**Keywords:** TGF-β superfamily, physiological oxygen, physoxia, chondrocytic marker expression, TGF-β receptors, type I, type II, type III, Sp1/3, *ALK1*, *ALK5*, betaglycan, endoglin

## Abstract

Culturing articular chondrocytes under physiological oxygen tension exerts positive effects on their extracellular matrix synthesis. The underlying molecular mechanisms which enhance the chondrocytic phenotype are, however, still insufficiently elucidated. The TGF-β superfamily of growth factors, and the prototypic TGF-β isoforms in particular, are crucial in maintaining matrix homeostasis of these cells. We employed a feedback-controlled table-top bioreactor to investigate the role of TGF-β in microtissues of human chondrocytes over a wider range of physiological oxygen tensions (i.e., physoxia). We compared 1%, 2.5%, and 5% of partial oxygen pressure (pO_2_) to the ‘normoxic’ 20%. We confirmed physoxic conditions through the induction of marker genes (*PHD3*, *VEGF*) and oxygen tension-dependent chondrocytic markers (*SOX9*, *COL2A1*). We identified 2.5% pO_2_ as an oxygen tension optimally improving chondrocytic marker expression (*ACAN*, *COL2A1*), while suppressing de-differentiation markers (*COL1A1,* *COL3A1*). Expression of TGF-β isoform 2 (*TGFB2*) was, relatively, most responsive to 2.5% pO_2_, while all three isoforms were induced by physoxia. We found TGF-β receptors *ALK1* and *ALK5* to be regulated by oxygen tension on the mRNA and protein level. In addition, expression of type III co-receptors betaglycan and endoglin appeared to be regulated by oxygen tension as well. R-Smad signaling confirmed that physoxia divergently regulated phosphorylation of Smad1/5/8 and Smad2/3. Pharmacological inhibition of canonical ALK5-mediated signaling abrogated physoxia-induced *COL2A1* and *PAI-1* expression. Physoxia altered expression of hypertrophy markers and that of matrix metalloproteases and their activity, as well as expression ratios of specific proteins (Sp)/Krüppel-like transcription factor family members SP1 and SP3, proving a molecular concept of ECM marker regulation. Keeping oxygen levels tightly balanced within a physiological range is important for optimal chondrocytic marker expression. Our study provides novel insights into transcriptional regulations in chondrocytes under physoxic *in vitro* conditions and may contribute to improving future cell-based articular cartilage repair strategies.

## 1. Introduction

### 1.1. Articular Cartilage and Hypoxia

Adult articular cartilage is avascular and chondrocytes are able to survive in this environment with a relatively low oxygen content. The oxygen gradient across articular cartilage tissue ranges from about 7% O_2_ in the superficial zone to less than 2% O_2_ in the deep zone [1,2,3]. It has been demonstrated that varying oxygen tensions (pO_2_) in cartilage can significantly influence chondrocyte metabolism, proteoglycan synthesis, and the levels of growth factors released by cartilage-derived cells (i.e., chondrocytes) [4,5]. Optimal physiological conditions to maintain chondrocyte homeostasis in vitro have been suggested to be between 2% and 5% pO_2_ [6,7]. Appropriate physoxia (i.e., physiological level of ‘hypoxia’) not only helps with promoting extracellular matrix (ECM) protein syntheses, but also with maintaining a proper phenotype and metabolism of these cells [8]. Hypoxia-inducible factor is an oxygen-dependent transcriptional activator that consists of a constitutively expressed HIF-1β subunit and one of three alpha subunits. Hypoxia-inducible transcription factors (HIF-1α), whose expression and function are mainly post-translationally regulated by hydroxylation reactions [9], was discovered by Semenza et al. HIF-1α is the major isoform for induction of hypoxic genes and often serves as an indicator protein [10,11,12], while the ‘real’ oxygen sensors are hydroxylases (i.e., prolyl-hydroxylases, PHD-1 to 3; reviewed by e.g., Semenza [13]). Under ambient oxygen concentration, these proteins have a very short half-life (<5 min) as Von Hippel–Lindau factors target them for degradation [13]. When oxygen level is low (1–5%), hydroxylation decreases and HIF-1α can escape the degradation, to heterodimerize with constitutive HIF-1β (ARNT) and to induce target gene expression.

### 1.2. TGF-β Signaling in Articular Cartilage

The diverse transforming growth factor (TGF-β) superfamily consists of 33 members, most of which are dimeric, secreted polypeptides, and includes, among others, bone morphogenetic proteins (BMPs) [14,15]. In articular chondrocytes, TGF-β superfamily signaling is important to homeostasis and development. TGF-β is able to induce SOX9 synthesis and to promote type II collagen and extracellular matrix (ECM) synthesis through activation of the SMAD2/3 phosphorylation pathway [16]. TGF-β signals through a pair of transmembrane serine/threonine kinases known as the TGF-β type II (TGFBRII) and type I (TGFBRI) or activin receptor-like kinases (ALKs) receptors. Here, the ligand usually binds a constitutively active kinase TGFBRII, which phosphorylates TGFBRI, resulting in activation of TGFBRI kinase activity [14,16]. Canonically, TGF-β ligands bind to the TGF-β type II receptor (TGFBRII) which, in most cells, then recruits the type I receptor activin-like kinase 5 (ALK5) to phosphorylate a cytoplasmic protein known as SMAD (small mothers against decapentaplegic) [17]. The intracellular effector molecules of ALK5 are transcription factors (TFs) SMAD-2 and -3, to exert transcriptional regulation [17].

Previously is was shown that, in addition to the canonical TGF-β-ALK5/Smad2/3 pathway, chondrocytes express another activate TGF-β type I receptor, ALK1, which activates Smad1/5 pathway. The TGF-β/ALK5 and TGF-β/ALK1 pathways exert opposing effects in chondrocytes [18]. It has been suggested that there is a shift from the canonical TGF-β signaling pathway to TGF-β /Smad1/5 signaling pathway during cartilage degeneration [19]. Altered ALK5/ALK1 ratios thus appear responsible for otherwise seemingly contradictory and context-dependent ambivalent actions of TGF-βs in these cells [12,19]. Factors shifting the balance between ALK5/Smad2/3 and ALK1/Smad1/5 signaling pathways in human chondrocytes remain incompletely understood. This balance between SMAD 2/3 and SMAD 1/5/8 signaling, which is activated by TGF-β isoforms and BMP ligands, plays a key role in cartilage homeostasis [20,21]. The three mammalian TGF-β isoforms (TGF-β1, -2, -3) are structurally related, pleiotropic cytokines encoded by separate genes with often similar autocrine or paracrine actions, among others, maintaining and regenerating adult tissues [22]. These prototypic members of the superfamily also play an indispensable role in cartilage repair and homeostasis [11,12], with often similar effects in vitro, but the three TGF-β isoforms also have non-redundant functions in vivo [14,15]. TGF-β is also an effective inducer of chondrogenesis [23] by stimulating chondrocyte proliferation, while inhibiting chondrocyte hypertrophy and maturation in vitro [24,25]. In contrast, BMP2 and BMP4, are potent regulators of chondrocyte hypertrophy and matrix degradation [26,27].

Others have reported that the increased ratio of ALK1 to ALK5 correlated with increased expression of catabolic markers in human and mouse OA chondrocytes [28,29,30]. Thus, it seems that activation of the ALK1 pathway promotes a de-differentiated, fibroblast-like, phenotype in chondrocytes, while activating the ALK5 route stimulates anabolic activities like TGF-β mediated ECM production, such as type II collagen. Determining factors that differentially regulate the balance between ALK5/Smad2/3 and ALK1/Smad1/5 signaling pathways will provide insights into the cellular mechanisms by which TGF-β regulates chondrocyte function.

### 1.3. Hypoxia and Chondrocyte Metabolism

Lafont et al. recently reported that hypoxia inhibits BMP-2-induced SMAD signaling in monolayer cultures of human articular chondrocytes [31], which indicates that hypoxia modulates the sensitivity of these cells for BMP signaling. In addition, it has been reported that the crosstalk between TGF-β and hypoxia signaling pathways regulates many physiological processes in various tissues [32]. Since TGF-β is known to induce *COL2A1* expression in chondrocytes, it is tempting to speculate that increased *COL2A1* expression in physoxic chondrocyte cultures in vitro may be caused by stimulated TGF-β signaling. Dissecting molecular mechanisms underlying physoxic induction of chondrocyte markers might aid in further improving cell-based chondral repair strategies, as well as improving chondrocyte culture conditions for research purposes.

In this research, we focused on TGF-β signaling during chondrocyte re-differentiation in cytokine-free 3D pellet cultures upon standard monolayer expansion [33,34]. As in vivo cartilage is exposed to a low oxygen tension, we studied effects of physiological oxygen tension on chondrocytes in a table-top bioreactor system [3,35,36], by comparing 1%, 2.5%, and 5% pO2, respectively, to normoxia (20%), as we hypothesized that a certain level of physoxia, between 1% and 5% pO_2_ may be optimal for cell homeostasis and ECM synthesis. We further hypothesized that TGF-β signaling might be divergently affected by different levels of physoxia, contributing to a balanced ALK1 vs. ALK5 receptor-mediated cell signaling.

## 2. Results

### 2.1. Bioreactor Validation

**Figure 1:**
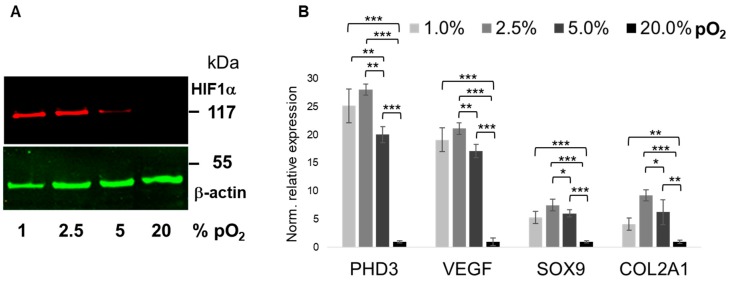
Validation of hypoxic conditions and its effect on marker gene expression. Figure shows representative HIF-1α protein expression next to that of β-actin as loading control (**A**). Relative mRNA expression of hypoxia- and chondrocyte-specific markers (**B**). As expression of all four markers was significantly suppressed under “hypoxic” conditions (≤5% pO_2_), as compared to “normoxic” conditions (i.e., 21% pO_2_), we confirmed robust oxygenation levels within the micro-bioreactor. Screening of marker gene expression after 48 h further revealed significant differences in mRNA levels between 1% and 5% of oxygenation and also between 2.5% and 5% pO_2_. Oxygenation levels are shown as grey scales of increasing intensities from 1% to 21%, respectively. Molecular weight (kDa) is indicated; Western blots (*n* = 3), RT-qPCR (*n* = 6); * *p* < 0.05, ** *p* < 0.01, *** *p* < 0.001.

To validate the bioreactor-based in vitro culture system, we confirmed pO_2_-dependent expressional changes of HIF-1α itself (Figure 1A) and that of PHD3 and VEGF, which were both reported earlier to be most responsive to low pO_2_ in this setting [3]. HIF-1α stabilization was virtually undetectable under 20% pO_2_, but protein abundance increased with reduced pO_2_ levels. Only modest HIF-1α abundance was detected at 5% pO_2_, but at 2.5% pO_2_ and below its expression was stable, with no significant differences between 2.5% and 1% pO_2_, respectively. The expression of both PHD3 and VEGF was significantly, 20- to 25-fold, induced under pO_2_ levels of 5% or less. Both genes were further slightly, but significantly, more upregulated under 2.5% as compared to 5% pO_2_ (Figure 1B). In contrast, at 1% pO_2_ expression of both was non-significantly reduced, as compared to 2.5% pO_2_. Cartilage-specific markers *SOX9* and *COL2A1* showed the same trend of pO_2_ level-dependency, albeit to a lesser extent (Figure 1B).

### 2.2. Oxygenation-Dependent ECM Marker Expression

**Figure 2:**
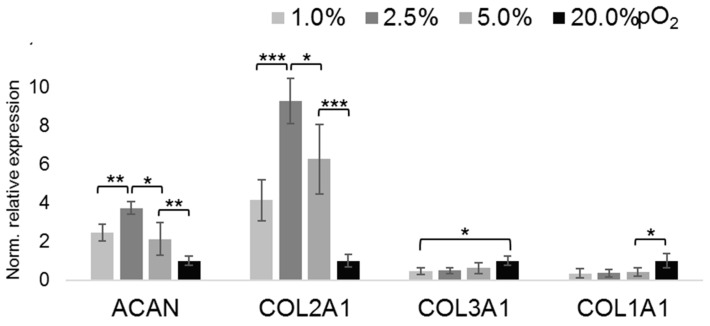
Oxygen tension-dependent expression of ECM markers. RT-qPCR gene expression analyses of aggrecan core protein (*ACAN*) and selected types of collagens as phenotypical markers to asses oxygenation level-dependent differentiation. Oxygenation levels (% pO_2_) appear as grey scales, as shown within the figure. *n* = 6; * *p* < 0.05, ** *p* < 0.01, *** *p* < 0.001.

To assess the effect of physoxia on the chondrocytic phenotype, we screened the expression of ECM markers associated with a differentiated (i.e., wanted) and de-differentiated (i.e., unwanted, fibroblastic) phenotype of articular chondrocytes. We used the expression of the core protein of aggrecan (*ACAN*), an important proteoglycan (PG) of the ECM of articular chondrocytes, and that of the structural key marker protein collagen type II (*COL2A1*) [37], as indicators of a proper phenotype (Figure 2). Expression of both genes showed an inverse pO_2_-dependency, up-regulation of mRNA abundances with down-regulated pO_2_ levels. In contrast, limited expression of fibroblastic differentiation markers *COL1A1* and *COL3A1* was either not significantly affected by the pO_2_-dose (*COL3A1*) or suppressed by physoxic pO_2_ levels (*COL1A1*) below 5%. In contrast, expression of chondrocytic markers *ACAN* and *COL2A1* was upregulated under lower oxygen levels, as compared to 20% pO_2_. Of note, the expression of both was relatively highest at 2.5% pO_2_.

### 2.3. Oxygenation-Dependent Expression of TGF-β Ligands

**Figure 3:**
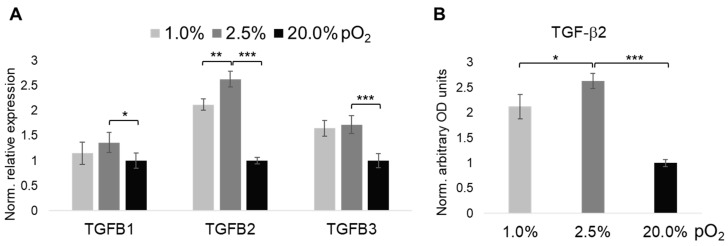
Oxygen tension-dependent expression of prototypic TGF-β ligand isoforms. We used RT-qPCR to screen oxygen-dependent changes in mRNA abundance of all three human TGF-β isoforms, *TGFB1-3* (**A**). As *TGFB2* was most prominently upregulated by extracellular oxygenation levels of 2.5% pO_2_, and below, we validated its expression on protein level (**B**), with its abundance in 20% pO_2_ set to 1. Oxygenation levels (% pO_2_) appear as grey scales as shown within the figure. RT-qPCR (*n* = 6), ELISA (*n* = 3); * *p* < 0.05, ** *p* < 0.01, *** *p* < 0.001.

The importance of the TGF-β superfamily for maintaining ECM homeostasis in articular cartilage encouraged us to screen the expression of all three human prototypic isoforms *TGFB1*, *TGFB2*, and *TGFB3*, respectively (Figure 3). While mRNA levels of *TGFB2* were, relatively, most prominently induced by 2.5% and 1% of physoxia, respectively, (Figure 3A), the other two isoforms were also both significantly up-regulated by lowering pO_2_ levels to 2.5%, as compared to 20%, with *TGFB3* being slightly more responsive than *TGFB1*. Induction of the most prominently regulated isoform, TGF-β2, by <2.5% pO_2_ was confirmed on the protein level (Figure 3B).

### 2.4. Oxygenation-Dependent Regulation of TGF-β Receptors

**Figure 4:**
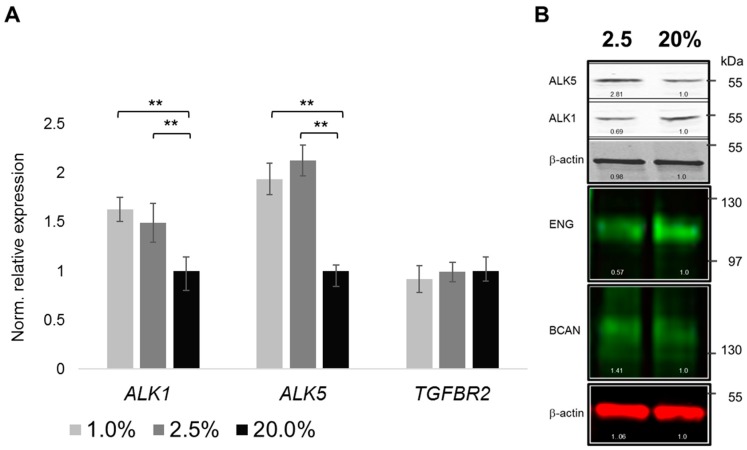
Oxygen tension-dependent abundances of TGF-β receptors. Normalized relative mRNA expression of TGF-β type I (*ALK1*, *ALK5*) and type II (*TGFBR2*) receptors (**A**). Representative images of TGF-β type I and type III (betaglycan, BGCN; endoglin, EGN) receptor protein abundances. Chondrocytes were cultured under 1% and/or 2.5% and 20% pO_2_, respectively; loading control, β-actin (**B**). MW in kDa on right. RT-qPCR (*n* = 5), immunoblots (*n* = 3); ** *p* < 0.01.

To facilitate TGF-β signaling, next to sufficiently abundant ligands, adequate expression of TGF-β receptors appears necessary. We, therefore, looked into potential pO_2_-dependent changes in the expression of canonical TGF-β signaling receptors, like type I receptors ALK1 and ALK5, as well as type II receptor TGFBRII. To this end, mRNA expression of *ALK1* and *ALK5* was up-regulated by physoxia (Figure 4A). *ALK5* expression was slightly more responsive than *ALK1.* TGFBRII mRNA (*TGFBR2*) abundance was not significantly altered by the chosen pO_2_ levels. Oxygenation-dependent regulation of type I receptors ALK1 and ALK5 was further verified on the protein level. Interestingly, a trend towards higher abundance of ALK5 under ≤2.5% pO_2_ levels, as compared to 20% pO_2_, was detected, while ALK1 showed the opposite trend. Finally, type III co-receptors (BGCN, ENG) may potentially modulate TGF-β signaling. While endoglin (ENG) expression was reduced under physoxia, faint betaglycan (BGCAN) immunoblotting signals appeared increased (Figure 4B).

### 2.5. Oxygenation-Dependent Regulation of R-Smad Phosphorylation

**Figure 5:**
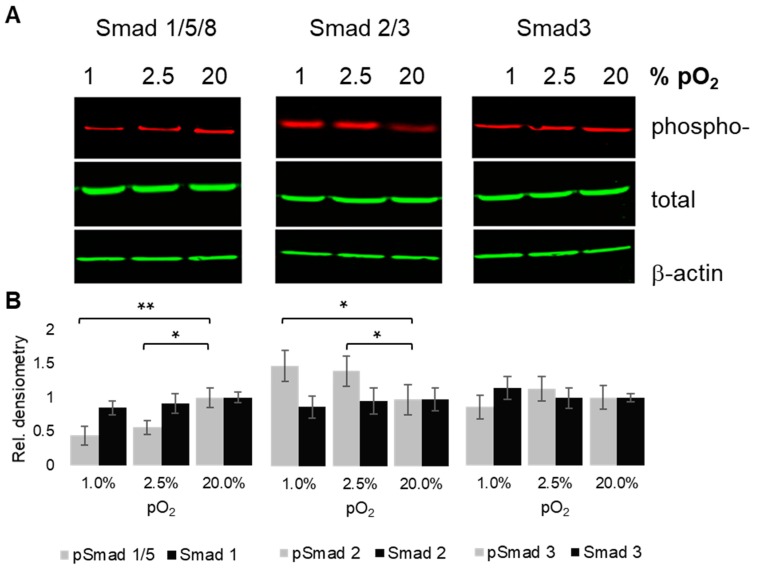
Oxygen tension-dependent R-SMAD phosphorylation. Chondrocytes were cultured under 1%, 2.5%, and 20% pO_2_, respectively. Immunoblots showing phosphorylated forms of Receptor SMADs 1/5/8, 2/3, and 3, respectively, next to their respective total expression levels (**A**); loading control, β-actin. Underneath: Densiometric signal quantification (**B**); triplicates from three donors; * *p* < 0.05, ** *p* < 0.01.

As an indication of altered TGF-β signaling, we further looked into oxygenation level-dependent changes of phosphorylation levels of Receptor SMADs (R-Smads). To this end, we used dual color immunoblotting to visualize phospho-SMADs in red and total SMAD levels in green (Figure 5A), with β-actin levels as internal loading controls. Overall, expression of total SMADs was relatively constant. While SMAD3 was not significantly differentially phosphorylated under conditions of low pO_2,_ as compared to normoxia, pSmad1/5/8 levels showed a decreasing trend with decreasing pO_2_. In contrast, Smad2/3 phosphorylation levels increased with decreasing oxygenation levels (Figure 5B).

### 2.6. Oxygen- and ALK-Dependency of The Articular Chondrocyte Phenotype

**Figure 6:**
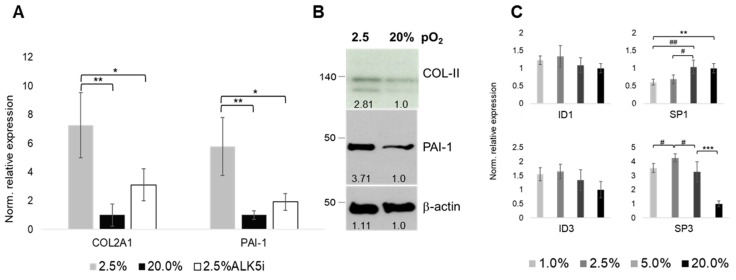
Oxygenation-tension and ALK5-dependent expression of collagen type II, next to PAI-1 and selected downstream targets ID and SP. Normalized relative mRNA expression of type II collagen (COL-II, i.e., *COL2A1*) next to that of ALK5-Smad2/3 downstream target *PAI-1* (plasminogen activator inhibitor-1). Pharmacological inhibition of ALK5 (ALK5i) suggests its involvement in *COL2A1* expression (**A**). Respective immunoblots showing protein abundances (**B**). Expression of ALK1-Smad1/5/8-target DNA-binding proteins inhibitor *ID1* and *ID3* and transcription factors *SP1* and *SP3*, respectively (**C**). Selected oxygenation levels were 2.5% and 20% pO_2_, respectively. RT-qPCR (*n* = 5), Western blot (*n* = 3); * *p* < 0.05, ** *p* < 0.01, *** *p* < 0.001.

We next used type II collagen (*COL2A1*) mRNA expression as a key indicator of a proper chondrocyte phenotype. Its expression was upregulated under physoxia (i.e., 2.5% as the optimal condition, see Figure 1 and Figure 2), as compared to 20% pO_2_, respectively. Pharmacological inhibition of ALK5 (ALK5i) partially reversed the physioxia-mediated *COL2A1* upregulation, being indicative of ALK5-mediated signaling underlying the *COL2A1* regulation (Figure 6A). Of note, expression of *PAI-1*, an established TGF-β responsive gene that involves ALK5-Smad2/3 signaling, is regulated in a similar way. Here, ALK5i also partially reversed the physioxia-mediated change in gene expression, being additionally supportive of the aforementioned notion. Oxygenation-dependent transcriptional changes could be confirmed on the protein level (Figure 6B). Interestingly, while expression of ID-family members of transcription factors were not significantly influenced by the applied pO_2_ levels, SP-family members SP1 and SP3, respectively, were oxygenation-dependent inversely regulated (Figure 6C). SP1 expression was decreased, while SP3 expression was increased by physoxia.

### 2.7. Oxygen-Dependent MAPK and MMP Activation

Finally, TGF-β signaling is known to be modulated by cross-talks with protein kinases, like mitogen-activated protein kinases (MAPKs). A recent study [31] focused on the BMP-driven regulation of *COL2A1* to investigate the role of p38 kinase in this context. We investigated the TGF-β-ERK (extracellular signal-regulated kinases) axis, as this was not addressed in the former study. As MAPKs further regulate matrix metalloproteases (MMPs) [38] and MMP-2 activity is hypoxia-dependent [39], we studied oxygen-dependent expression of *MMP-13* and *COL10A1* as hypertrophy markers and general gelatinolytic activity.

**Figure 7:**
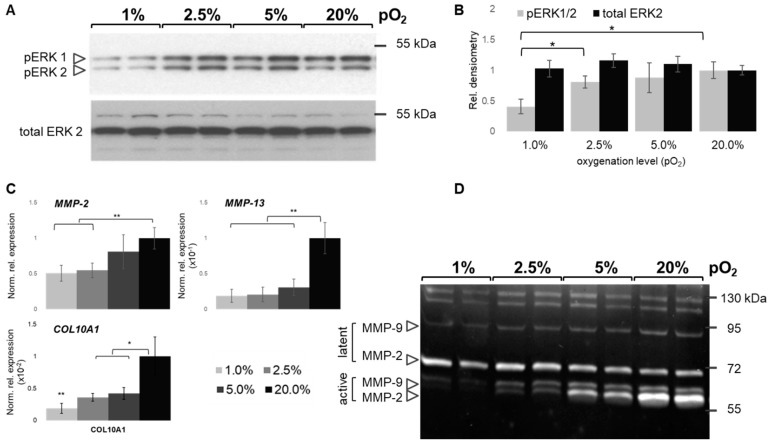
Oxygen-dependent regulation of extracellular signal-regulated kinase (ERK) and matrix metalloprotease (MMP) activity. Representative Western blots showing phosphorylated active ERK (pERK1/2; p44/42, respectively) and total ERK2 as loading control from two donors (**A**) next to densiometric signal quantification (**B**). Normalized relative gene expression levels of *MMP-2* and selected hypertrophy markers *MMP-13* and COL10A1 (**C**). Gelatin zymography (**D**) shows latent MMPs and respective gelatinolytic activity of active forms, mainly derived from gelatinases MMP-2 and MMP-9. MW, kDa, on right. Oxygen percentages (pO_2_) as gray scales (light gray 1%, black, 20%). Representative images derived from pooled pellets are shown. RT-qPCR (*n* = 4), Western blot (*n* = 3); zymogram (*n* = 2); * *p* < 0.05, ** *p* < 0.01.

ERK activation appeared to be inversely dependent on pO_2_ (Figure 7A). Interestingly, 1% pO_2_ revealed limited ERK phosphorylation by relatively constant total ERK abundance, as compared to the other conditions, and ERK activation is, thus, suppressed under sever hypoxia as compared to normoxia. Interestingly, pERK levels did not significantly differ between 2.5%, 5%, and 20% pO_2_, while below 2.5% its activation is suppressed (Figure 7B). Low oxygen levels reduce hypertrophy marker expression in chondrocytes as evident from mRNA expression of *MMP-13* and *COL10A1* (Figure 7C). *MMP-2* expression showed the same trend. To this end, its mRNA abundance is in line with the activity profile (Figure 7D), which reveals a reduced activity at ≤2.5% as compared to higher pO_2_ values. Activity of both human gelatinases, MMP-2 and MMP-9, appears significantly reduced at ≤2.5%.

## 3. Discussion

### 3.1. Physoxia-Preserved Chondrocyte Phenotype

A sophisticated micro-bioreactor enabled us to tightly control dissolved oxygen levels (i.e., partial oxygen pressure; pO_2_) to keep the culture medium within a narrow range of 2–5% for evaluating effects of physoxia on 3D cultured human articular chondrocytes. Often, extreme values were compared, like 1% vs. 21% [31,40], or 5% vs. 21% [41], or 3% vs. 20% [3]. We now identified 2.5% pO_2_ to optimally support expression of several chondrocytic key markers.

Interestingly, at 5% pO_2_ we found HIF-1α expression to be half-maximal, which is indicative of its raised degradation speed [3] and is in line with our expectations, while at ≤2.5% its expression appeared stable. Upregulation of hypoxia-inducible genes, like *PHD3, VEGF*, *SOX9*, and *COL2A1* [3], confirmed this notion.

To the best of our knowledge our approach has not been reported earlier with human primary articular chondrocytes. While several past studies aimed at mimicking physiological oxygen levels experienced by articular chondrocytes in situ in experimental settings in situ, few screened over a wider range of pO_2_ and apparently missed subtle differences in marker gene expression. In addition, generally, 2D culture has been used to evaluate effects of ‘hypoxia’, which is a limitation [42]. In contrast, optimal physoxia suppressed several undesired markers of hypertrophic or fibroblastic differentiation, like *COL1A1*, *COL3A1*, and *COL10A1* or *MMP-13*. This is in line with earlier findings also reporting that HIF-1α inhibits fibroblast-like markers *COL1A1* and *COL3A1* during hypoxia-induced chondrocyte re-differentiation [41], when comparing 2D and 3D cultures of human articular chondrocytes.

Importantly, physoxia affects the expression of human TGF-β isoforms slightly differently, as follows: While TGF-β1 was least affected, TGF-β2 and -β3 were positively physoxia-responsive. Remarkably, TGF-β2 mRNA abundance was most highly induced by 2.5% pO_2_, which could be confirmed on protein level. Earlier, potent chondrocytic effects of TGF-β, especially at low concentrations (1ng/mL), were shown to be dependent on low oxygen levels of <5% [43]. It is important to realize that TGF-β isoform 2 is a more potent stimulator of type II collagen synthesis than TGF-β1 [3,44]. TGF-β2 is further unique among the human isoforms in that it lacks an RGD integrin-binding sequence in its precursor and the only isoform containing multiple CRE elements in its promoter [45]. Of note, the CREB family of transcription factors, binding to these elements, are hypoxia-responsive in other cell types. Moreover, in other cells, CREB-1 decreases profibrotic type I collagen expression through TGF-β signaling [46], which may partially explain further suppression of *COL1A1* levels at ≤ 5% pO_2_ in our study. That physoxia increases general TGF-β levels, and in some cells, particularly TGF-β2 [47], is also in line with earlier studies demonstrating positive effects of hypoxia on TGF-β level.

In another tissue context, *PAI-1* induction through HIF-1α-mediated TGF-β upregulation was reported [48]. Thus, consistent with previous reports, ’hypoxia’ increased ECM protein abundances, such as key marker collagen type II and *ALK5* target *PAI-1*, and is in line with low oxygen tension, providing more favorable conditions for maintaining a proper chondrocyte phenotype [6,7].

Recently, it was reported that endoglin (i.e., CD105) can differentially regulate TGF-β1-induced Smad2/3 and Smad1/5 signaling in order to balance ECM production in human chondrocytes [49], in normoxic conditions. Our results now indicate that this may hold for TGF-β2 under ‘hypoxic’ conditions. In their different setting, endoglin inhibited TGF-β1-induced *COL2A1* and *PAI-1* expression and we, rather, observed a down-regulation of endoglin by 2.5% pO_2_. We did not stimulate with TGF-β2 and cultured in 3D, which could account for discrepancies between both studies, as endoglin expression also depends on the chondrocyte differentiation state and increases with passage numbers. Of note, endoglin binds TGF-β1 and TGF-β3, but not TGF-β2 [50], distinguishing it from betaglycan. Hypoxia increased endoglin mRNA and protein expression, while it is co-regulated by p38, and perhaps JNK [51]. Parker et al. [52] showed heteromeric endoglin:betaglycan complexes in human chondrocytes, occurring in a ligand- and TGFBRII-independent manner. This suggests an even more complex interdependent regulatory TGF-β signal transduction network. TGF-β2 lacks affinity to types I and II receptors in chondrocytes in the absence of betaglycan, which appears upregulated by physoxia, in the present study. Surprisingly, TGF-β2 exhibits a high affinity to this type III receptor that, in turn, facilities its binding to TGFBRII [52]. Although expression of the latter receptor was not apparently altered by oxygen levels, it is intriguing to speculate about a regulatory feedback loop in which hypoxia stimulates betaglycan and TGF-β2 synthesis to improve Smad2/3 signaling through ALK5, which is induced under these conditions, too. Ultimately, this can improve expression of down-stream targets, like *COL2A1*. As endoglin can inhibit SMAD2 phosphorylation, its suppression under physoxia may remove a potential block from canonical the ALK5-Smad2/3 signaling route. Of note, endoglin and ALK1 were reported to act in the same pathway in chondrocytes [49]. In a variety of other human cell types, TGF-β and hypoxia together transcriptionally enhance endoglin expressions [53], pointing to cell-type specific differences. Our data indicate a suppressive effect of physoxia on ALK1-Smad1/5 signaling and endoglin expression. For chondrocytes in hypoxic 3D culture, TGF-β2 appears to be the more important isoform, which cannot bind to endoglin. Although further research is needed, our data potentially adds another exciting aspect to the puzzle of how hypoxia may modulate TGF-β signaling to improve the chondrocyte phenotype in situ.

### 3.2. Regulation of Collagen and MMP Expression

True collagenases (i.e., MMP-1, -3, -13) are important ECM remodeling enzymes and MMP-13, in particular, is considered primarily catabolic in cartilage. Oxygen-dependent MMP activation, under 5% and 21%, was earlier reported [54], but only MMP-2, and not -9, mRNA was down-regulated under lower oxygen tension. Zymography data were also not unequivocally supportive of the semi-quantitative mRNA analyses. In contrast, our data show a down-regulation of both gelatinases, and their respective activities, at lower oxygen tension. Earlier, about the same level of ‘hypoxia’ downregulated matrix metalloproteases (MMPs) on the mRNA and protein level [38] and suppressed MMP-2 activity [39]. Our data further confirm oxygen-dependent expression of *MMP-13* and *COL10A1*, two hypertrophic markers that are unwanted when aiming at generating stable hyaline cartilage. In the axolotl, Smad2 is responsible for the action of TGF-β during regeneration, whereas Smad3 is not required, and Mmp2 and Mmp9, respectively, were identified as Smad2 target genes, whereas Mmp13 appeared to be a non-canonical TGF-β target [55]. Thus, during some regenerative cellular programs, Smad2 and Smad3 may be differentially regulated by TGF-β and Smad2 activating may be relatively more important. As hypoxia is a general stress signal during regenerative processes, conserved cell signaling patterns may explain why we observed a relatively more prominent reaction of SMAD2, than of SMAD3, to physoxia.

The transcriptional regulation of hypoxia inducible factor has been reviewed elsewhere [11] and both, HIF1A and HIF2A are subject to a plethora of oxygen-independent and dependent post-transcriptional modifications. While HIF1A contains an ERK1 recognition site, HIF2A does not [11], which may hint towards divergent transcriptional control. We found ERK activation to be inversely dependent on pO_2_. Extensive cross-talks between MAPKs, and ERK in particular, have been reported from other tissues [56,57]. MEK/ERK activation by TGF-β also has been reported for articular cartilage [58]. Noncanonical TGF-β activating kinase 1 (TAK1) signaling activates several MAP kinases (MAPKs), including p38, JNK, and ERK [59] and at least affects the Smad1/5/8 route and, thus, canonical and noncanonical BMP pathways. TGF-β induced activation of p38 or ERK1/2 is furthermore essential for transcriptional activation of Smad2 and Smad4, and, thus, for maximal activation of specific Smad-dependent transcriptional responses, in murine chondrocytes [59].

Lafont et al. recently reported hypoxic BMP-2 induction of *COL2A1* through p38 action [31], while mRNA levels of BMP receptors were unaltered by hypoxia alone. The authors did not study TGF-β ligands. In agreement with our data, the unstimulated condition showed slightly more pSmad1/5/8 under 21% than under 1% of oxygen, too. Thus, while signaling through the BMP-p38 axis under hypoxia appears to contribute to ECM marker expression, co-contribution of canonical TGF-β signaling, as suggested by our data, cannot be excluded. To this end, it is intriguing that most BMPs, and BMP-2 in particular, are usually associated with induction of hypertrophic differentiation and may be least suitable to maintain an articular chondrocyte phenotype [60,61] in regenerative settings. Interestingly, under normoxic conditions in murine chondrocytes, prototypic TGF-β signaling was reported to regulate phosphorylation and stabilization of the Sox9 protein, and, thus, likely also increases *COL2A1* through p38- and Smad-dependent mechanisms, independently [62]. Other studies suggest that betaglycan’s primary function in TGF-β signaling may be to “present” ligands, such as TGF-β2, to the type II TGF-β receptor to activate downstream signaling. Sometimes, betaglycan’s core domain, however, may also regulate BMP signaling through directly binding BMP-2, although with lower affinity as compared to TGF-β [63]. Membrane-bound betaglycan can further enhance TGF-β2 signaling to promote TGF-β signaling, while secreted betaglycan may suppress TGF-β/BMP signaling [64]. We did not study the BMP-Smad axis or specific details of type III receptor signaling, which is a limitation of the present study towards drawing clear conclusions into this direction.

Molecular regulation of collagen expression is complex. As endoglin splice variants may opposingly effect the ALK1-Smad1-*ID1* and ALK5-Smad2-*PAI-1* route [65], in other cells, we looked at the hypoxic regulation of selected transcription factors. Helix-loop-helix transcription factors Id1 and Id3 contribute to controlling cell division in human chondrocytes [66] and are expressed in re-differentiating chondrocyte pellet cultures [67]. Id1 was further identified as TGF-β/ALK1/Smad1 target gene in hepatic stellate cells [68], but both were not significantly regulated in this study.

In contrast, transcription factors Sp1 and Sp3 were. Both of which are able to enhance or repress promoter activity [69]. SP3/SP1 ratios are important regulators of the proximal *Col10a1* promoter in hypertrophic cartilage and holds the potential to regulate a broad variety of other collagen types during chondrocyte differentiation [70]. TGF-β-downregulation of human type II collagen in articular chondrocytes involves SP3/SP1 ratios [71] and further depends on cooperation of Sp1 with other transcription factors [72]. HIF-1α is able to down-regulate type I collagen expression through SP3 in human chondrocytes [73], which is in line with our initial ECM marker profiles under physoxia. Interestingly, Sp1, which is downregulated by TGF-β1, is further involved in the repression of TGF-β type I and type II receptors, (TβRI) and type II (TβRII), respectively, and hypoxic upregulation of SP3 may thus further facilitate TGF-β signaling. Of note, Duval et al. suggested a model in which, under hypoxia, Sp3 outcompetes Sp1 to act as a strong inhibitor of *COL1A1* transcription [73]. Our data further confirm their trends in gene regulation of hypertrophy markers *MMP-13* and *COL10A1* [39] by oxygen. Markway et al. compared normal to osteoarthritic chondrocytes under 2% and 21% pO_2_ and, while the cell source largely determined expression of *COL1A1, COL10A1, MMP-2,* and *MMP-13* per donor group, all genes revealed largely the same oxygen-dependent trend in regulation. Of note, TGF-β signaling is known to be altered in osteoarthritic cartilage [28,29,30] and it would be interesting to also compare a broader range of physoxic responses of chondrocytes derived from healthy to those from OA cartilage in the future.

### 3.3. Regulation of TGFβ Signaling Balance under Physoxia

Physoxia increases ALK5 mRNA and protein levels, while ALK1 mRNA abundance is much less regulated and ALK1 receptor abundance seems suppressed at 2.5% pO_2_. Earlier, the canonical TGF-β pathway, ALK5-Smad2/3, was reported to promote ECM synthesis by chondrocytes. In contrast, the same study reported ALK1-Smad1/5 signaling to inhibit ECM synthesis [18]. This is in agreement with a report of ALK5-Smad2/3 signaling activation suppressing chondrocyte differentiation [74]. Increasing ALK1/ALK5 ratios seem to be detrimental to cartilage, elevating e.g., MMP-13 expression, and to cause osteoarthritis-like symptoms in mice and men [20]. In our hands, physoxia decreased ALK1-Smad1/5/8 signaling activity, while promoting that of ALK5-Smad2/3. The latter is in good agreement with increased collagen type II production and the promotion of chondrocyte metabolism and cartilage homeostasis through canonical TGF-β signaling. Recently, Van Caam et al. [75] even demonstrated that TGF-β-induced SMAD2/3 and SMAD1/5 phosphorylation may both be ALK5-kinase-dependent in primary chondrocytes. Unfortunately, currently available small molecule inhibitors are not able to distinguish both ALK-mediated pathways [75] and our findings that ERK1/2 activation is suppressed only indicate a potential fine-tuning of this non-canonical TGF-β-MAPK pathway through physoxia.

Optimal physoxia further appears to suppress SMAD1/5/8 phosphorylation, while it activates SMAD2/3 (mainly SMAD2). Our findings contribute to a better understanding of the divergent role ALK1- and ALK5-mediated signaling in health, aging, and disease [29], even though our in vitro study knows several limitations and cannot fully reflect in vivo conditions.

To this end it is interesting that, within the physoxic range, the expression of most chondrocytic, anabolic genes peaks at 2.5% pO_2_, as compared to 1% or 5%, respectively. Chondrocytes’ glycolytic pathway of energy generation seems to control mainly cell proliferation, while mitochondrial oxidative phosphorylation appears responsible for tissue regeneration [76]. Upregulation of ECM markers under physoxia suggests that differentiation/repair processes may be predominant, rather than proliferation, and thus may at least partially explain this phenomenon, which requires more research for its clarification. Direct comparison of multiple defined physoxic oxygenation levels in parallel were rarely performed in the past. As reactive oxygen species (ROS) can affect chondrocyte metabolism [77,78] through their release from mitochondria under severely anoxic conditions [7], we studied a moderate range of oxygen tensions. Physoxia around 2–3% appears to robustly induce HIF-1α, while being above the level that seems to decrease ATP production in chondrocytes [5].

In summary, our data strongly suggest that physoxia-induced secreted endogenous TGF-β2 efficiently stimulates Smad2/3 phosphorylation. A novel finding of our study is that ECM marker gene expression is relatively highest at 2.5% pO_2_ and it would be interesting to study other collagen types and chondrocytic marker genes in the future. We contributed novel insights on how physoxia, as an important environmental factor in cartilage, differentially regulates ALK1-Smad1/5/8 and ALK5-Smad2/3 signaling pathways divergently in human chondrocytes in vitro. Furthermore, we identified the SP-family of transcription factors as candidates enhancing hypoxia-driven synthesis of ECM markers, like *COL2A1*. The detailed identification of an optimal range of oxygenation for in vitro may provide critical insights into novel mechanisms by which TGF-β regulates chondrocyte function.

## 4. Materials and Methods

### 4.1. Chondrocyte Expansion Culture

Human cartilage biopsies from up to six patients, depending on the experiment (details in figure legends), with gonarthritis undergoing total knee replacement surgery (ethics committee approval ID: MEC08-4-028, MEC2004-322) were used for this study. Details on tissue harvesting, digestion, and cell seeding were earlier reported by us [3]. After five days of pre-culture, spherical chondrocyte microtissues were cultured in a Pall Micro-24 MicroReactor system (MRT-24; Pall Corp., Port Washinton, NY, USA), while Das et al. [3,79] reported culture conditions.

### 4.2. Bioreactor Culture System and ALK5 Inhibition

We described the prototype of the MRT-24 system used to automatically feedback control oxygen gradient cultures, ranging from 1% to 20% pO_2_, earlier in more detail [3,35,36]. Briefly, six microtissues were cultured per well of a customized 24-well MRT-24 cassette, in four milliliters of DMEM medium, as described recently [3]. Briefly, oxygen tension (pO_2_) was lowered by continuous sparging with nitrogen and then automatically maintained at the desired set-point (either 1%, 2.5%, 5%, or 20%) by using air as a counter gas.

Pharmacological intervention was essentially performed as reported earlier by us [3] using an SB-525334 (Sigma-Aldrich Chemie GmbH, 91625 Schnelldorf, Germany), but now 15 µM and 2.5 h prior to induction of physoxic conditions.

### 4.3. RNA Isolation and RT-qPCR Experiments

Briefly, microtissues were homogenized using QIAshredder (Qiagen, 40724 Hilden, Duitsland; #79654), according to the manufacturer’s instructions, to resuspend 0.75 million cells in 350 µL RNABee (TEL-TEST Inc., Friendswood, TX, USA), with the addition of chloroform and 20 min at 13,000× *g* [3]. Total RNA was then isolated from the supernatant using a Qiagen RNA Micro Kit, according to the instructions (Qiagen GmbH, Hilden, Germany), and the nucleic acid content was determined spectrophotometrically (NanoDrop^®^ND1000, Isogen Life Science, IJsselstein, The Netherlands). In compliance with MIQE guidelines [80], good RNA integrity (RIN ≥ 8.5; BioAnalyezr 2100, Agilent Technologies, Amstelveen, Netherlands) and the absence of PCR inhibitors was ensured [3,81]. Reverse transcription was performed using the RevertAid^TM^ First Strand cDNA Synthesis Kit (Thermo Scientific, Rockford, IL, USA) with mixed priming (oligo-d(T):random hexamers, 3:1, v/v), according to the supplier’s instructions. Both cDNA synthesis and the RT-qPCR method, with specificity and efficiency controls as well as data normalization to a reference gene index, were described in detail elsewhere by us [3]. Briefly, the geometric mean of CT-values of pre-evaluated endogenous calibrators ubiquitin’(UBC), beta-actin (ACTB), hypoxanthine phosphoribosyltransferase1 (HPRT1), and RPLP0 [3] was used to normalize expression data. The “best house-keeper index” was chosen [3] and normalized relative fold-change in gene expression was calculated according to the 2^−∆∆*C*t^ method [82]. Averaged replicates per patient and condition were used to calculate the relative expression, normalized to the 20% control.

Individual differences were first averaged and replicates then used to calculate the relative expression, normalized to the internal control. RT-qPCR primers for extracellular matrix (ECM) components (*ACAN, COL1A1, COL2A1, COL3A1*), sex determining region Y-box 9 (*SOX9*), markers of hypoxia like prolyl hydroxylase 3 (*PHD3*) or vascular endothelial growth factor (*VEGF*), TGF-β isoforms, TGF-β receptors (activin-like receptor kinases (*ALK1, ALK5*), *TGFBR2*, betaglycan (*BGCAN*) and endoglin (*EGN*), as well as TGF-β signaling target (plasminogen activator inhibitor-1 (*PAI-1*) were previously referenced by us [3], together with the cycling protocol,. RT-qPCR assays for MMPs [83], primers to detect human inhibitors of DNA binding (ID1, ID3) [3], human SP1 and SP3 [84] can be found elsewhere. Newly adopted assays [31] are as follows: RPLP0 5′-CCTAAGGCAGGAAGATGGGGTG-3′, 5′-AGTCTGCTTGTACCCCAGGA-3′; COL10A1 5′-CAAGGCACCATCTCCAGGAA-3′, 5′-AAAGGGTATTTGTGGCAGCATATT-3′; SMAD1 5′-CTACCCTCACTCTCCCACCA-30 50-GCACCAGTGTTTTGGTTCCT-3′, SMAD5 5′-TCGAAGAGGATTGTAATCATGG-3′; 5′-CCTACAGTGCAGCCACTAGC-3′; ID-3 5′-CTGGACGACATGAACCACTG-3′, 5′-GTAGTCGATGACGCGCTGTA-3′.

### 4.4. Protein Isolation, Immunoblotting, and ELISA

Protein isolation of membrane receptors was essentially performed as described earlier [3]. Briefly, tissue spheroids were harvested in a hypotonic PEC homogenization buffer and homogenized using Eppendorf^TM^ autoclavable safe-lock micro-pestles (Fisher Scientific, 63505 Langenselbold, Germany; #10683001) in the presence of cOmplete^TM^ Mini protease inhibitor (Roche Diagnostics, 1322 CK Almere, Netherlands) and phosphatase inhibitors (Sigma-Aldrich, Cocktail 3, #P2850) cocktails. Handlings were at 4 °C with cells and devices pre-chilled on ice. Cells were homogenized (RW20, IKA Werke GmbH & KG, 79219 Staufen im Breisgau, Germany) and extracts successively centrifuged at 4 °C from 10 min at 700× *g* to 90 min at 100,000× *g* to obtain membrane fractions [85]. Protein purification from cytoplasmic cell lysates was described elsewhere by us [as were sample purification, bicinchoninic acid (BCA)-based protein quantification assay, separation by SDS-polyacrylamide gel electrophoresis (PAGE), and electro-blotting techniques. Ordering information of, and concentrations for, anti-type II collagen and anti-PAI-1 antibodies were also reported in Das et al.

The primary antibodies that were used for this study include pSmad1 (9511), pSmad2 (3101), pSmad3 (9520),Smad2 (3103, Cell Signaling, 1:1000); ALK5 (Santa Cruz, 1:1000); ALK1 (R&D system; 1:500); HIF1-α (BD Biosciences, 1:1000); Smad1 (Zymed, 1:1000); Smad 3 (Abnova 1:1000); PAI (BD Biosciences 1:1000); type II collagen (Chemicon 1:1000); and endoglin (Santa Cruz P4A4, 1:1000). Occasionally the membranes were stripped with Restore^TM^ Western Blot Stripping Buffer (Thermo Scientific, #21062), according to the manufacturer’s instructions, and then reprobed with β-actin (Santa Cruz, 1:1000) antibodies as a loading control.

Chemiluminescent detection [86,87] and dual color detection using infrared-labeled probes on an Odyssey imaging system (Li-Cor Biosciences; 61352 Bad Homburg vor der Höhe, Germany) were earlier reported by us [88]. Visualization was then performed by using IRDye 680RD- and IRDye 800CW-conjugated secondary antibodies (1:15,000; both from Li-Cor Biosciences). Replicate data were normalized to β-actin (1:1000; Cell Signaling Technology) as a loading control. Signal intensities were quantified using ImageJ software (National Institutes of Health; Sacaton, AZ 85147, USA).

### 4.5. ELISA and Zymography

Ordering details and instructions for using the TGF-β2-specific ELISA assay can be found elsewhere [37]. Briefly, medium was collected and centrifuged at 1200 rpm for 8 min to remove cell debris. The cleared supernatant was collected and stored at −80 °C, until further use. TGF-β2 concentration was quantified spectrophotometrically using an enzyme-linked immunosorbent assay (ELISA; Demeditec Diagnostics GmbH, Kiel, Germany). TGF-β2 concentrations in the samples were calculated from a standard curve at OD450 nm, according to manufacturer’s instructions.

Zymography, as a means to detect oxygenation-dependent gelatinase activity (i.e., MMP-2 and -9, respectively), was described earlier by us [89]. Briefly, secreted MMPs were determined in YM-30 (Millipore, Billerica, MA, USA) concentrated conditioned culture medium. Zymogram loading buffer (BioRad, Hercules, CA, USA) was added and incubated for 10 min at 37 °C. Electrophoretic separation in gelatin gels (BioRad) was done at 100 V, 25 mA. MMPs were renatured using Triton-X100 buffers exactly as described [89]. Gels were stained and de-stained using Coomassie brilliant blue and Roti destain (both Carl Roth, Karlsruhe, Germany), respectively, to visualize proteolytic activity halos against a dark background of undegraded gelatin.

### 4.6. Statistical Analysis

Statistical analysis was performed using SPSS 13.0 software. We used the replicate raw expression data from multiple donors and tested for the effect of our experimental conditions using a mixed-effects linear regression model. We, therefore, accounted for the correlation in the data that exists within each donor. We further incorporated “donor” as a random effect to correct for basal differences in expression between patients [33]. A value of *p* < 0.05 was considered significant and histograms represent means ± SD and are reported as significance levels * *p* < 0.05, ** *p* < 0.01, and *** *p* < 0.001.

## 5. Conclusions

**Figure 8:**
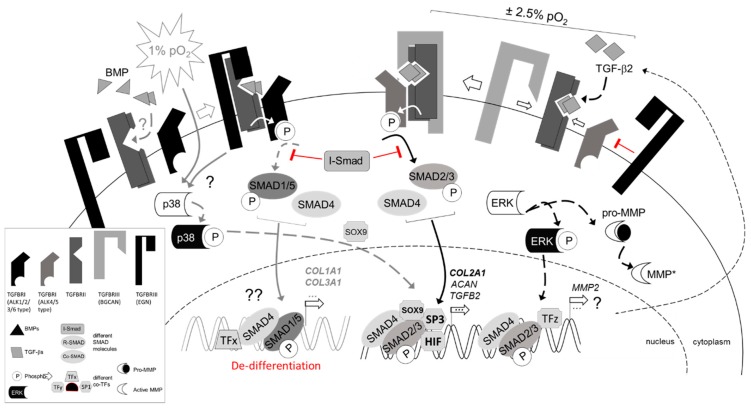
Simplified schematic of proposed TGF-β signal transduction under physoxic conditions. TBSF signaling is mediated via specific heteromeric complexes of type I (activin A receptor type II-like kinases, ALKs) and type II (TGFBRII) serine/threonine kinase receptors. Canonically, TGF-β interacts with TGFBRII and ALK5, but in some cells (e.g., chondrocytes) signaling through ALK1 may also occur [29]. Physoxia prominently upregulates TGF-β2, while severe hypoxia was also reported to result in nuclear SOX9 accumulation upon BMP-2 stimulation in a p38 MAPK-dependent, but Smad-independent manner [31]. This is shown on the left in gray, as it was not studied here. BMPs commonly signal through BMPRII, ActRIIA, or ActRIIB as type II receptors and type I receptors ALK1, 2, 3, and 6. Co-receptors (TGFBRIII) betaglycan and endoglin can modulate TGFBRII/ALK5 and TGFBRII/ALK1 signaling in a cell type-dependent manner. Intracellular signaling can be divided into two main Smad-mediated signaling pathways. ALK1, 2, 3 and 6 induce phosphorylation of Smad1, 5 and 8, while ALK5 induces phosphorylation of Smad2 and Smad3, respectively. Activated R-Smads form heteromeric complexes with common Smad4 to translocate into the nucleus, where they can act as transcription factors (TF) in complexes together with gene specific co-TFs, like ID or SP molecules, to regulate expression of specific target genes. The ratio of these co-TFs may fine-tune gene expression of, for example, collagen molecules. Physoxia stimulates TGF-β2 and its co-receptors BGCAN, while suppressing EGN. To this end, physoxia seems to stimulate ALK5-mediated signaling pathways, shown on the right. Putative involvement of ERK1/2 and MMP-2 is indicated. Arrows (black) indicate stimulation (gray, putative), while blocks (red) indicate inhibitory effects. Please note: To simply the cartoon, only ALK1 and ALK5 were used to represent both pathways, type II and type III receptors are not illustrated as dimers, and soluble co-receptors, reported in other cells, were excluded. [31]

We show that physoxia increases the expression of TGF-β2, betaglycan, and ALK5 and Smad2/3 phosphorylation, while it decreases levels of ALK1, Smad1, phospho-Smad1/5, and endoglin in human chondrocytes. Moreover, physoxia dose-dependently regulates transcription factors to enhance ECM protein production, like SP1/SP3. Taken together, as summarized in a simplified cartoon (Figure 8), we identified 2.5% of physoxia as optimal for key markers expression and show that physoxia is a critical regulator of TGF-β signaling and ECM synthesis in human chondrocytes. We further demonstrate that hypoxia enhances production of ECM proteins, such as type II collagen and PAI-1. We suggest that this may primarily occur due to endogenously stimulated TGF-β2, in response to physoxia.

## Abbreviations

MDPI	Multidisciplinary Digital Publishing Institute
ECM	Extracellular Matrix
TBSF	TGF-β superfamily
TF	Transcription factor
